# ‘Give ‘em the vape, sell ‘em the pods’: razor-and-blades methods of pod e-cigarette pricing

**DOI:** 10.1136/tobaccocontrol-2020-056354

**Published:** 2021-03-25

**Authors:** Harry Tattan-Birch, Jamie Brown, Sarah E Jackson

**Affiliations:** Behavioural Science and Health, University College London, London, London, UK

**Keywords:** tobacco industry, electronic nicotine delivery devices, advertising and promotion, price

The razor-and-blades model is a pricing strategy of selling base products, like razor handles, at a loss but making profits on repeated sales of complementary goods, like blades (reflected by the proverb ‘Give ‘em the razor, sell ‘em the blades’, widely misattributed to King C. Gillette).^1^ This strategy has been used across a myriad of industries, from games consoles to inkjet printers.^1^ More recently, it has been adopted by pod electronic cigarette (‘e-cigarette’) manufacturers.

**Figure 1:**
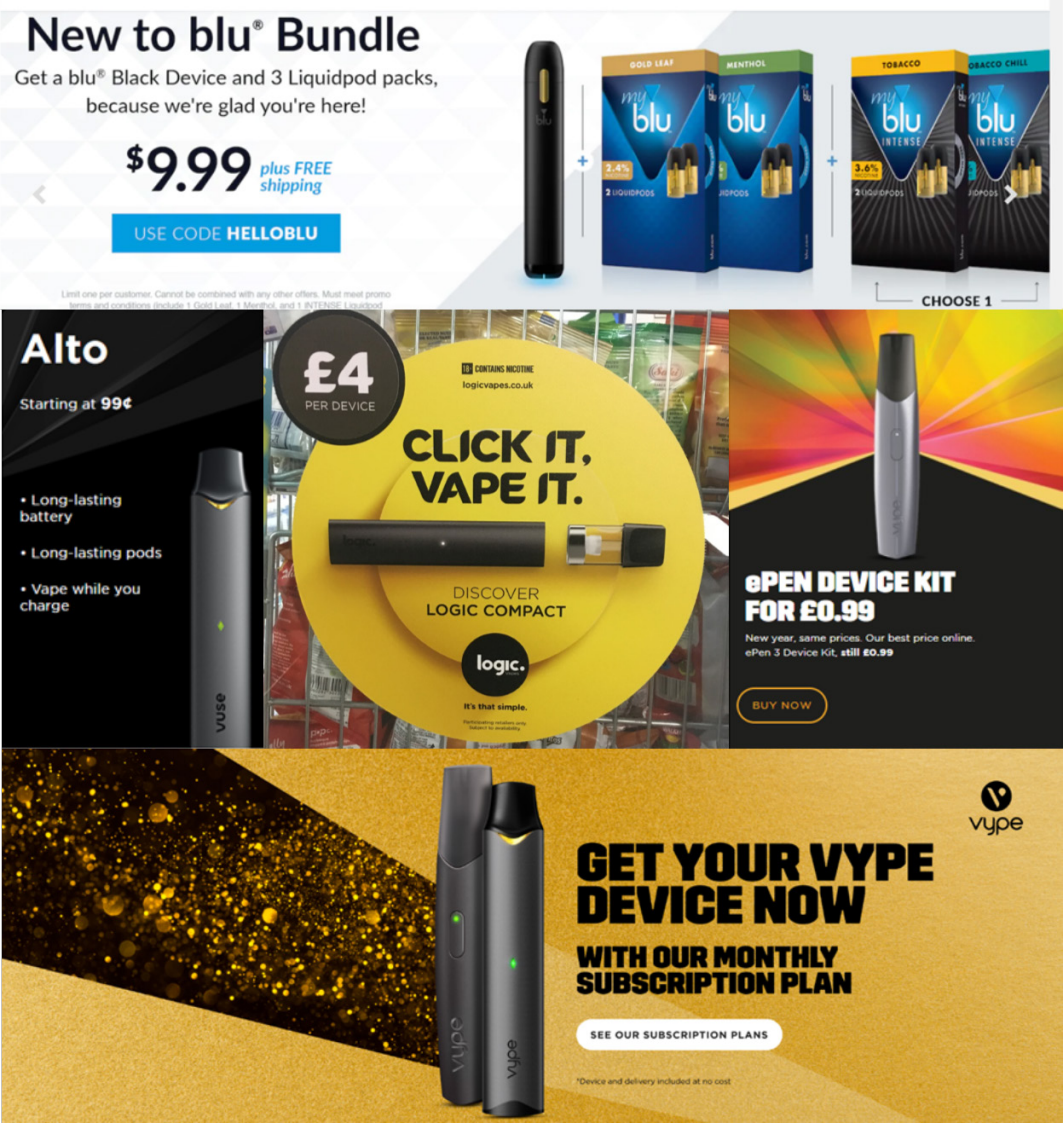
(Top) New customer deal on the blu US online store, offering an e-cigarette and six pods for only US$9.99. (Middle-left) Alto pod e-cigarette priced at US$0.99 on the Vuse US online store. (Middle-centre) Point-of-sale advertisement for the Logic Compact, available for £4 (US$5.5) at convenience stores in the UK. (Middle-right) ePen pod e-cigarette priced at £0.99 on Vype’s UK website. (Bottom) Promotion for Vype’s ePen, which is available for free in Canada when users sign up for a monthly subscription to pods. This promotion was also found on billboard advertisements in the UK.

Pod e-cigarettes like JUUL, Vuse, blu, and Logic use disposable cartridges (‘pods‘) that are pre-filled with e-liquid. On average, these cartridges cost four times the price of the same amount of bottled e-liquid, making them more expensive in North America than the equivalent number of combustible cigarettes.^2^ So how do pod e-cigarette manufacturers overcome this price differential? Across North America and Europe, some have begun using razor-and-blades pricing models — providing a base e-cigarette device (‘vape’) cheaply or for free (figure 1) but making large profits on disposable device-specific pods.^1 3^

This strategy may influence both uptake of vaping and the types of devices vapers choose, with potential behavioural, public health, and economic implications. First, it could encourage smokers to try switching to vaping. One of the key barriers that stops smokers from using e-cigarettes is the perceived cost of the devices.^4 5^ Greater availability of devices with no or low upfront cost might attract smokers to try e-cigarettes as a way to stop smoking cigarettes — which could have a positive impact on public health.^6^ But, once they start vaping with razor-and-blades priced devices, the increased cost of continued pod use might reduce the number of smokers switching completely compared with other e-cigarettes or nicotine products.^4^ Alternatively, the increased cost of pod use might encourage users to switch to refillable e-cigarettes, which can be topped up cheaply with bottled e-liquid.^7^

Second, it might encourage uptake of vaping among young people. The recent rise in youth vaping in the US has been driven by increased use of pod devices.^8^ Just as cigarette singles and 10 packs were used to attract young people with little disposable income to smoking,^9^ the low upfront cost of razor-and-blades priced pod devices may have contributed to rises in youth vaping seen in some countries, such as the US. In the UK, there has been recent evidence of British American Tobacco distributing Vype for free without age verification, which resulted in people under 18 receiving free samples.^3^ Surprisingly, the activity is not illegal as of October 2020 due to a loophole in the regulatory framework. The loophole requires urgent attention given the UK has thus far succeeded in relatively low youth uptake of e-cigarettes, especially among those who have never smoked.^10^ The success may be attributable to careful regulation, which included an early ban on advertising that could cross borders and sale of the products to children.^11^

Thirdly, it may draw vapers away from refillable e-cigarettes towards pod devices.^12^ As refillable e-cigarettes can be filled with any generic brand of e-liquid, manufacturers cannot make profit by offering these devices for free or selling them at a loss. This could shift the market in favour of e-cigarette brands that are owned by tobacco companies: the most popular pod systems are at least partially owned by tobacco manufacturers, while independent retailers are more likely to sell refillable devices.^13^ A shift towards tobacco industry-owned products brings with it the risk that profits made could be used to fund lobbying efforts or expansion into markets in low- and middle-income countries.^14^ Moreover, unlike independent e-cigarette manufacturers, tobacco companies are incentivised to encourage — or at least be ambivalent about — dual use of e-cigarettes and combustible cigarettes rather than complete substitution.

Finally, it could widen smoking-related economic inequalities. Already, smokers from socio-economically disadvantaged groups spend a much higher proportion of their income on cigarettes.^15^ The low upfront cost of razor-and-blades priced pod e-cigarettes may attract people from these groups, despite the total cost of continued use vastly exceeding that of refillable devices.^16^ Since disadvantaged individuals are more likely to continue vaping after they quit smoking cigarettes,^17 18^ increased use of pod devices could place an even greater economic burden on disadvantaged individuals.

Pricing strategies are just one driver of consumer demand for different nicotine products. Other factors, like worsening perceptions about e-cigarette harm relative to cigarettes and COVID-19 related vape shop closures, also likely play a role.^7 19 20^ Nonetheless, there is work to be done exploring the effects of razor-and-blades tactics on device choice, youth vaping and e-cigarette use for smoking cessation. If it is apparent that these tactics are boosting the market share of tobacco industry-owned e-cigarettes or encouraging youth vaping, policymakers might consider marketing or pricing restrictions.
